# Changes in the Biotransformation of Green Tea Catechins Induced by Different Carbon and Nitrogen Sources in *Aspergillus niger* RAF106

**DOI:** 10.3389/fmicb.2019.02521

**Published:** 2019-11-01

**Authors:** Xiang Fang, Minru Du, Tong Liu, Qian’an Fang, Zhenlin Liao, Qingping Zhong, Jianwen Chen, Xiaolin Meng, Shiyu Zhou, Jie Wang

**Affiliations:** Guangdong Provincial Key Laboratory of Nutraceuticals and Functional Foods, College of Food Science, South China Agricultural University, Guangzhou, China

**Keywords:** green tea catechins, *Aspergillus niger*, tannase, monooxygenase, dioxygenase, radical-scavenging activity, carbon and nitrogen sources

## Abstract

Biotransformation of green tea catechins mediated by microbes and/or enzymes could increase their bioavailability and improve their health benefits, but the regulatory mechanism remains unclear. Here, *Aspergillus niger* RAF106 isolated from Pu-erh tea was proved to be capable of degrading gradually ester-catechins into non-ester-catechins with higher bioavailability and gallic acid (GA) in aqueous solution only containing green tea catechins, and the products displayed similar radical-scavenging activity *in vitro* with the control. Meanwhile, the degradation was mediated by inducible enzymes as the extracellular form, and tannase might be an important enzyme among the extracellular enzymes. Moreover, it was found for the first time that the biotransformation was accelerated significantly by the addition of different nitrogen sources (i.e., yeast extract, peptone, NaNO_3_, and NH_4_Cl) and lactose through stimulating the hyphal growth and the tannase activities but was inhibited by glucose effect. Furthermore, nitrogen sources continued to promote the degradation of GA and/or non-ester-catechins by up-regulating the transcriptional expression of two dioxygenases and 10 monooxygenases. Besides, the addition of different nutrient sources except yeast extract did not alter the radical-scavenging activity of green tea catechins during the whole fermentation. These results provide a global insight into the roles of *A. niger* RAF106 and different nutrient sources in mediating the biotransformation of green tea catechins and modifying the radical-scavenging activity of biotransformed catechins.

## Introduction

Tea, one of the most popular beverages worldwide, is rich in polyphenols, known as green tea catechins, consisting primarily of four components: (-)-epicatechin (EC), (-)-epigallocatechins (EGC), (-)-epicatechin gallate (ECG), and (-)-epigallocatechin gallate (EGCG) (Reygaert, 2014). Green tea catechins were gained wide attention due to their antioxidant, antimicrobial, antimutagenic, and anticarcinogenic properties (Graham, 1992; Cao and Ito, 2004; Majchrzak et al., 2004). Also, they are associated with reduced risks for several diseases, such as cancer, coronary heart diseases, arteriosclerosis, and type two diabetes (Guo et al., 1996; Vita, 2003; Hou et al., 2004; Rizvi et al., 2005). However, less than 5% of the orally administered dose of green tea catechins reached the systemic circulation in rat and approximately 1.68% of ingested catechins were present in human’s plasma (0.16%), urine (1.1%), and feces (0.42%) after tea ingestion over 6 h (Warden et al., 2001; Catterall et al., 2003; Lin et al., 2007). Their low bioavailability was an important factor leading to the inconsistencies observed between *in vitro* and *in vivo* studies or between laboratory tests and epidemical studies (Cai et al., 2018).

Biotransformation using enzymes or whole-cell microbes may provide an effective solution by modifying the structures of green tea catechins while maintaining and even improving their original bioactivities (Gupta et al., 2013). Intestinal bacteria degraded ester-catechins to non-ester-catechins which were then degraded into hydroxyphenyl-γ-valerolactones, smaller phenolic acids and other metabolites, and the degradation improved the bioavailability of green tea catechins by more than 32.1%, showed anti-oxidative, anti-proliferative and anti-inflammatory activities, and enhanced CD4^+^ T cell activity as well as natural killer cell activity (Takagaki and Nanjo, 2010; Takagaki et al., 2011, 2014; Chen and Sang, 2014; Kim et al., 2016). Green tea catechins modified by lactic acid fermentation enhanced the overall antioxidant capacity and increased cellular uptake of tea catechins (de Lacey et al., 2014; Zhao and Shah, 2016). Enzymes, like tannase and hydrolase, could convert EGCG and ECG to EGC and EC, respectively, and biotransformed green tea catechins exhibited a significantly higher antioxidant activity, strongly inhibited the formation of *N*-nitrosodimethylamine and decreased the toxicity of EGCG without affecting the anti-proliferative effects (Lu and Chen, 2008; Zhong et al., 2008a, b; Macedo et al., 2012; Ni et al., 2015; Liu et al., 2017; Ong and Annuar, 2017). However, compared with the microbial transformation, the enzymatic strategy employs the enzymes isolated from microbes and the strict control of catalytic reaction, which might lead to the increase in the use of toxic chemicals and energy (Gupta et al., 2013).

*Aspergillus niger* has been generally regarded as a safe material by FDA and developed in some scaled-up processes to produce different enzymes (Ni et al., 2015). It has been reported that *A. niger* could degrade tea polyphenols of Pu-erh tea (*Camellia assamica*) in a solid fermentation system, and *A. niger* tannase and/or hydrolase had been applied in the transformation of green tea catechins and improved their antioxidant activities (Zhong et al., 2008b; Qin et al., 2012; Ni et al., 2015). However, no additional studies have been investigated on the regulatory mechanism of the biotransformation process mediated by *A. niger*, though studies concerning regulatory aspects of tannase production by molds in submerged and solid-state fermentation showed that tannase titers were associated with tannic acid, some of its derivatives and glucose (Bajpai and Patil, 1997; Bradoo et al., 1997; Aguilar et al., 2001). This study seeks to explore the roles of *A. niger* RAF106 isolated from Pu-erh tea in the biotransformation of green tea catechins in the media only containing green tea catechins, and to elucidate the effects of different nutrients on the biotransformation process, related enzyme activities, transcriptional expression of mono-/di-oxygenases, and antioxidant capacities for the first time. Our results provide an insight into roles for *A. niger* RAF106 and different nutrient sources in mediating the biotransformation of green tea catechins, producing biotransformation-related enzymes, and sustaining the antioxidant capacities of green tea catechins, which will be helpful to further illuminate the metabolic pathway and metabolic modulation mechanism on microbial biotransformation of green tea catechins and pave the way for industrial implement.

## Materials and Methods

### Chemicals and Reagents

Green tea catechins were obtained from Jiangxi Lvkang Natural Products Co., Ltd., Yichun, China. Standard polyphenol compounds [EC, EGC, (-)-gallocatechin (GC), ECG, EGCG, (-)-gallocatechin gallate (GCG), and gallic acid (GA)] were purchased from Sigma-Aldrich (St Louis, MO, United States). Chromatographic grade methanol was bought from Hanbon Science and Technology (Jiangsu, China). All the other reagents used were of analytical grade.

### Fungi and Identification

One gram of Pu-erh tea was washed with 10 mL of sterile saline, and then diluted to 10^–1^, 10^–2^, 10^–3^, and 10^–4^ of levels. The dilutions were, respectively, incubated on potato dextrose agar (PDA) with 1% green tea catechins to isolate fungi. The extracted DNA of the isolated fungus was subjected to the amplification of ITS1 region fragment for identification, and then ITS1 sequence was registered in the NCBI.

The major fungus isolated was identified to be *A. niger*, and named with *A. niger* RAF106 (CGMCC NO.9608), according to its morphological characteristics and ITS1 sequence (MN195121). The fungus was stored at −80°C.

### Culture Conditions

*Aspergillus niger* RAF106 was cultivated in PDA at 30°C for 5 days to collect the conidia. 500 μL of a 10^8^ conidia/mL suspension was added into 100 mL of distilled water with 12 g/L green tea catechins (TC medium), and incubated at 30°C for 120 h by shaking at 150 rpm for biotransformation. 1.8 mL of supernatant were taken for HPLC at 0, 12, 16, 20, 24, 36, 48, 60, 72, 96, and 120 h of incubation.

### Quantification of Green Tea Catechins by HPLC

A 20 μL aliquots of the samples (100 × dilution) filtered through membrane filters (0.45 μm) were injected into an HPLC system equipped with a Phenomenex Luna C18 column (150 × 4.6 mm, 5 μm) and a UV detector for analysis of EGCG, EGC, ECG, EC, GCG, GC, and GA. The samples were eluted with a gradient system consisting of solvent A [methanol/formic acid/distilled water (9:0.5:90.5, v/v)] and solvent B [methanol/formic acid/distilled water (80:0.5:19.5, v/v)] delivered at a flow rate of 1 mL/min with UV detection at 270 nm. The gradient was run from 0 to 32% solvent B in 30 min, and the column temperature was maintained at 30°C. The concentration of phenolic compounds was determined by using external calibration curves of the standard compound at different concentrations.

### Effect of Induction on Transformation Enzymes From *A. niger* RAF106

To investigate the effect of induction on transformed enzymes, *A. niger* RAF106 was cultivated in PDB (PDA without agar, uninduction medium) and PDB supplemented with 12 g/L green tea catechins (induction medium) for 24, 48, 72, and 96 h, respectively. After cultivation, cultured broth was centrifuged at 4°C and the supernatant was collected and filtered through a 0.22 μm pore filter to be served as crude extracts of extracellular enzymes for the biotransformation of green tea catechins, while fungal cells were ground in liquid nitrogen and suspended in acetate buffer (200 mM, pH 5.2) as crude extracts of intracellular enzymes for the biotransformation of green tea catechins. The enzymatic reaction was carried out according to Zhong et al. (2008a). Briefly, the reaction mixture consisting of 2 mL of 12 g/L green tea catechins, 0.2 mL of crude enzyme sources and 3.8 mL of acetate buffer were incubated with shaking at 40°C for 12 and 24 h, respectively. The enzyme control and the substrate control consisted of 0.2 mL of crude enzyme sources and 3.8 mL of acetate buffer, and 2 mL of 12 g/L green tea catechins, 0.2 mL of deionized water and 1.8 mL of acetate buffer, respectively. HPLC was applied to analyze the content of EGCG, ECG, and GCG in the samples. Efficiency of *X* degradation (%) = (1–final *X*/original *X*) × 100, *X* means EGCG, ECG or GCG.

### Tannase Assay

Tannase activity was tested according to the spectrophotometric method of methanolic rhodanine (Sharma et al., 2000). Briefly, the substrate solution (0.01 M propyl gallate suspended in 0.05 M citrate buffer, pH 5.0), cell-free supernatant, and citrate buffer were pre-incubated at 30°C for 10 min before the enzyme reaction. The reaction mixture containing 0.25 mL of the substrate solution, 0.25 mL of the cell-free supernatant and 0.3 mL of methanolic rhodanine (0.667%; w/v) was kept at 30°C for 5 min, and then supplemented with 0.2 mL of 0.5 N potassium hydroxide. After incubated at 30°C for 5 min, each tube was diluted with 4.0 mL distilled water and incubated at 30°C for 10 min. The control tube was added again with 0.25 mL of the cell-free supernatant at this moment and the blank tube was added with 0.25 mL of citrate buffer instead of the cell-free supernatant in the reaction mixture. The absorbance was read against water at 520 nm using a Multiskan GO Reader (Thermo Fisher Scientific, Waltham, MA, United States). The enzyme activity was calculated by the formula: Δ*A*_520_ = (*A*_test_ − *A*_blank_) − (*A*_control_ − *A*_blank_). One unit of tannase activity was defined as micromole of GA formed per minute under described assay conditions.

### DPPH Assay

The scavenging activity of the stable 1,1-diphenyl-2-picrylhydrazyl (DPPH) free radical was used to assess the potential antioxidant capacity of green tea catechins and biotransformed green tea catechins according to the previous method (Macedo et al., 2012). Briefly, 2 mL of properly diluted samples and 2 mL of the DPPH working solution was mixed and incubated for 30 min in the dark. The absorbance at 570 nm was measured against a mixture of 2 mL of methanol and 2 mL of DPPH working solution as blank. The measurements were performed in triplicate.

### Effects of Nutrients on the Biotransformation Performance of *A. niger* RAF106

The effects of nitrogen were determined by adding 1 g/L nitrogen sources in the form of peptone (total nitrogen ≥14.0%, amino nitrogen ≥3.5%, burning residue ≤10.0%, and dry-weight loss ≤6.0%), yeast extract (total nitrogen ≥10.0–12.5%, amino nitrogen ≥5.1%, and sodium chloride ≤0.3%), NaNO_3_, or NH_4_Cl. The effects of carbon were determined by adding 60 g/L carbon source in the form of glucose, lactose or sucrose.

A 500 μL of a 10^8^ conidia/mL suspension was added into 100 mL of TC medium and TC medium supplemented with different carbon and nitrogen sources, and the cultures were incubated at 30°C for 120 h by shaking at 150 rpm. Then the process of green tea catechins determination by HPLC was performed as described above.

### RNA Isolation and qRT-PCR

Total RNAs were extracted from *A. niger* RAF106 grown in TC medium with/without extra NaNO_3_, NH_4_Cl, peptone, or yeast at 30°C for 48 h by shaking at 150 rpm using RNAiso^TM^ Plus Reagent (TaKaRa, Dalian, China) and transcribed reversely into cDNA with PrimeScript^®^ RT reagent kit (TaKaRa). Each cDNA were used as templates to analyze the transcriptional expression of tested genes via qRT-PCR with paired primers with SYBR^®^ Premix Ex Taq^TM^ (TaKaRa) (Supplementary Table S1). The relative transcription level of each gene was defined as the fold changed ratio of its transcript of cells obtained from TC medium with a certain nitrogen source over that from TC medium using the 2^–ΔΔCt^ method (Livak and Schmittgen, 2001).

### Data Analysis

Results from replicates were expressed as mean ± standard deviation (SD) and statistical analysis was subjected to one-factor analysis of variance performed with the Data Processing System (DPS) software. It is considered to be statistically significant when *p* < 0.05 in all experiments.

## Results

### Conversion of Ester-Catechins Into Non-ester-catechins Mediated by *Aspergillus niger* RAF106

**FIGURE 1:**
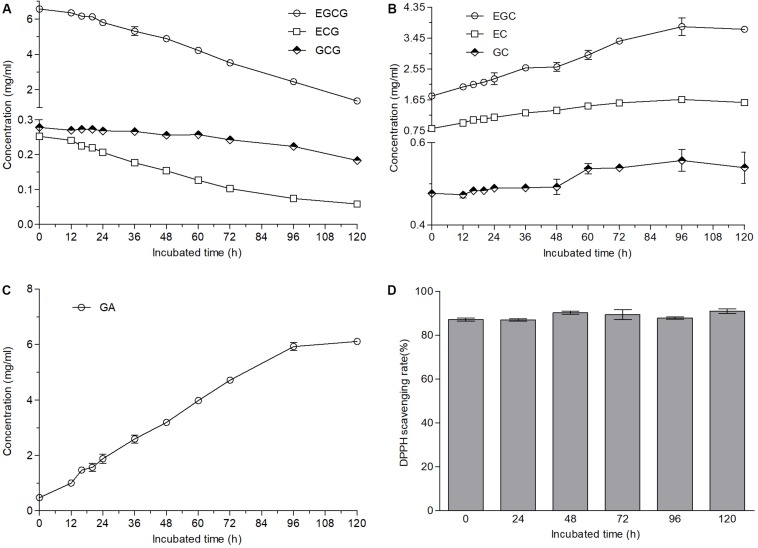
Changes in contents and antioxidant capacities of green tea catechins solutions after *A. niger* RAF106 was cultivated in the solutions only containing green tea catechins for 120 h. **(A–C)** The contents of ester-catechins, non-ester-catechins, and GA, respectively, in the supernatant of the cultures from different incubated hours. **(D)** DPPH scavenging rate of the supernatant of the cultures from different incubated hours.

The fungus grown well on the plates containing green tea catechins was identified as *A. niger* according to ITS1 sequencing and named as *A. niger* RAF106. Ester-catechins decreased but non-ester-catechins and GA increased when green tea catechins were incubated with *A. niger* RAF106 for 120 h (Supplementary Figure S1). As shown in Figure 1, the contents of ester-catechins including EGCG, ECG, and GCG were gradually decreased by fermentation of *A. niger* RAF106 in the liquid medium only containing 12 g/L green tea catechins. After 120-h fermentation, the levels of EGCG, ECG, and GCG were decreased by 79.1, 76.7, and 34.2%, respectively, accompanied with the increase of EGC, EC, and GC by 110, 92.7, and 13.1%, respectively (Figures 1A,B), as well as the increase of GA by 11.8 folds (Figure 1C). Meanwhile, EGCG, ECG, or GCG incubated with *A. niger* RAF106 was transformed into EGC, EC, and GC, respectively, except GA (data not shown). Moreover, DPPH assay showed that the supernatant fraction of green tea catechins incubated with *A. niger* RAF106 for different hours exhibited similar radical-scavenging activity with that of green tea catechins (Figure 1D), while the supernatant fraction of *A. niger* RAF106 could not scavenge the radical DPPH. The changed trends suggested *A. niger* RAF106 could acclimatize well itself to living in the media only containing green tea catechins and degrade ester-catechins into non-ester-catechins and GA but do not change the radical-scavenging capacity of green tea catechins after incubated with green tea catechins.

### Effect of Induction on Biotransformation Enzymes of Green Tea Catechins

**FIGURE 2:**
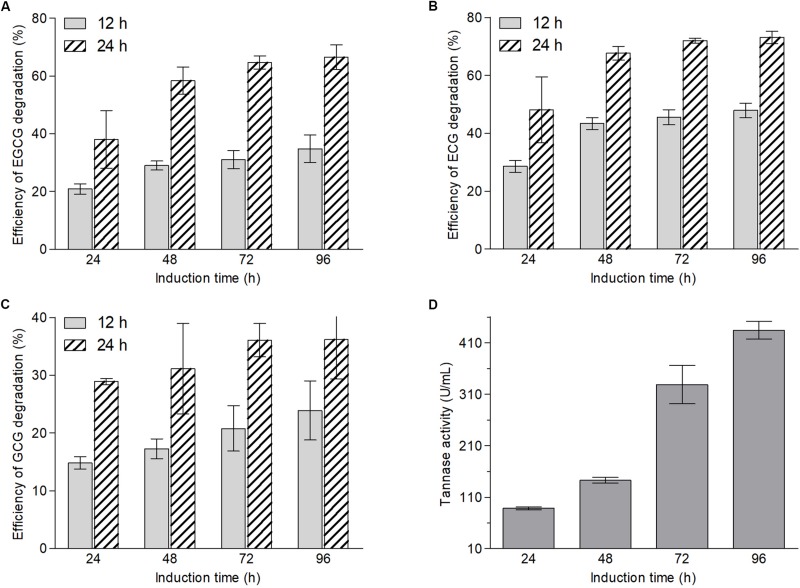
Changes in contents of EGCG **(A)**, ECG **(B)**, and GCG **(C)** after green tea catechins were incubated with crude enzyme sources collected from different induction hours for 12 and 24 h, respectively, and the tannase activities in different crude enzyme sources **(D)**.

According to the structures of ester-catechins and non-ester-catechins, ester-catechins are molecules with their corresponding non-ester-catechins as a core and the hydroxyl group in the C ring of non-ester-catechins esterified with GA. It is reported that the release of GA from ester-catechins depends on tannases/hydrolases, which are inducible enzymes transforming the gallate esters of tannins and other phenolic compounds, such as epigallocatechin gallate, into GA (Zhong et al., 2008a, b; Macedo et al., 2012; de Felipe et al., 2014; Yao et al., 2014). Therefore, to explore whether biotransformation enzymes of green tea catechins were inducible in *A. niger* RAF106, an induction test and an enzymatic degradation test using *A. niger* RAF106 from induced and uninduced cultures were conducted and the efficiency of EGCG, ECG, and GCG degradation was used to evaluate the catalytic capability of the crude enzyme sources. The concentrations of EGCG, ECG, and GCG were stable over a 24-h incubation period in the uninduced crude extracts from extracellular and intracellular enzymes (data not shown), however, the amount of EGCG, ECG, and GCG in the induced crude extracts from extracellular enzymes dropped significantly. Compared with the efficiency of ester-catechins degradation after 24 h of induction, the efficiency increased by 39.1, 48.9, and 66.8% for EGCG, 51.7, 59.3, and 67.7% for ECG, and 16.6, 40.2, and 61.4% for GCG after 48, 72, and 96 h, respectively, when the reaction time was 12 h, while the estimates increased by 53.7, 70.1, and 74.9% for EGCG, 40.7, 49.5, and 52.0% for ECG, and 7.7, 24.8, and 25.2% for GCG after 48, 72, and 96 h, respectively, when the reaction time was 24 h (Figures 2A–C). Meanwhile, compared with the efficiency of ester-catechins degradation after 12 h of reaction, the estimates increased 82.3–108.3% for EGCG, 52.5–68.3% for ECG, and 51.3–95.0% for GCG, respectively, after 24 h of reaction (Figures 2A–C). These data revealed the enzymes involved in the degradation of ester-catechins into non-ester-catechins and GA mediated by *A. niger* RAF106 were not constitutive but inducible extracellular ones, and the catalytic capability of inducible enzymes was positively relative to the induction time and reaction time in a certain range.

Moreover, tannase activities in different induced crude extracts of extracellular enzymes were assayed to testify whether tannases in *A. niger* RAF106 take part in the biotransformation of green tea catechins. The tannase activities displayed that the estimates increased by 61.3% after 48 h, 271.0% after 72 h, and 390.3% after 96 h, respectively, compared with that after 24 h of induction (Figure 2D). The trends of the tannase activities coincided with the changes in the contents of ester-catechins caused by enzyme crude sources, suggesting that tannases should be importance enzymes during the biotransformation of ester-catechins in green tea catechins into non-ester-catechins and GA mediated by *A. niger* RAF106.

### Effects of Carbon and Nitrogen Sources on the Biotransformation of Green Tea Catechins

To improve the biotransformation efficiency of green tea catechins mediated by *A. niger* RAF106, the medium only containing green tea catechins (TC medium) was modified by supplement with different carbon or nitrogen sources.

**FIGURE 3:**
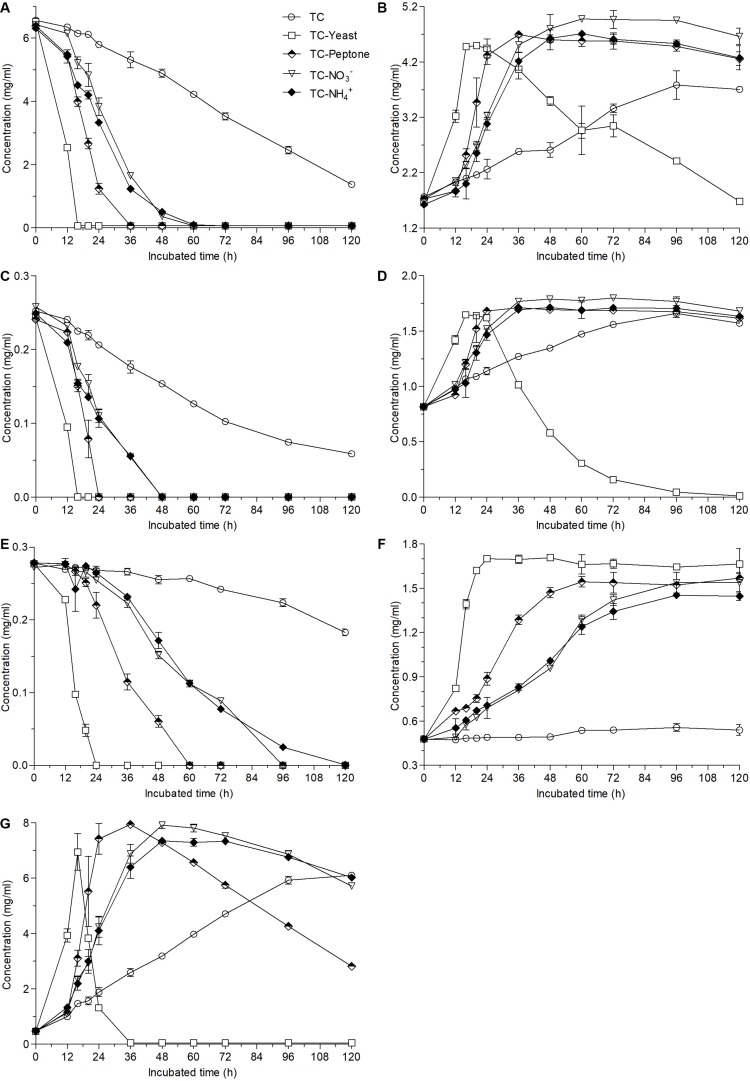
Changes in the contents of EGCG **(A)**, EGC **(B)**, ECG **(C)**, EC **(D)**, GCG **(E)**, GC **(F)**, and GA **(G)** caused by the addition of different nitrogen sources (1 g/L) into 12 g/L green tea catechins co-incubated with *A. niger* RAF106 for 120 h.

Compared with the biotransformation in the TC medium where less than 80% ester-catechins were converted into non-ester-catechin after fermentation for 120 h (Figures 1A–C), the contents of EGCG and ECG were nearly undetected at the 16th hour and GCG was also undetected at the 24th hour after fermentation of *A. niger* RAF106 in TC medium supplemented with yeast extract (Figures 3A,C,E). When peptone was added into TC medium, the fermentation time for undetectable EGCG, ECG, and GCG was 36, 24, and 60 h, respectively (Figures 3A,C,E). Meanwhile, EGCG and ECG were completely degraded at 60- and 48-h fermentation, respectively, when TC medium was mixed with NaNO_3_ or NH_4_Cl, and the concentration of GCG was lowered to 0 at 96-h incubation for NaNO_3_ and by 92.6% at 96-h incubation for NH_4_Cl (Figures 3A,C,E). The degradation of ester-catechins resulted in the accumulation of non-ester-catechins and GA, which was up to the maximum concentration at the time for the corresponding ester-catechins undetected (Figures 3B,D,F,G). Moreover, yeast extract accelerated the degradation of EGC, EC, and GA after long-term fermentation, and peptone, NaNO_3_ and NH_4_Cl also promoted the degradation of GA with the rate lower than that in yeast extract after long-term fermentation (Figures 3B,D,G). At the same time, it was found that only when GA was nearly degraded completely, EGC and EC began to be degraded (Figures 3B,D,G).

**FIGURE 4:**
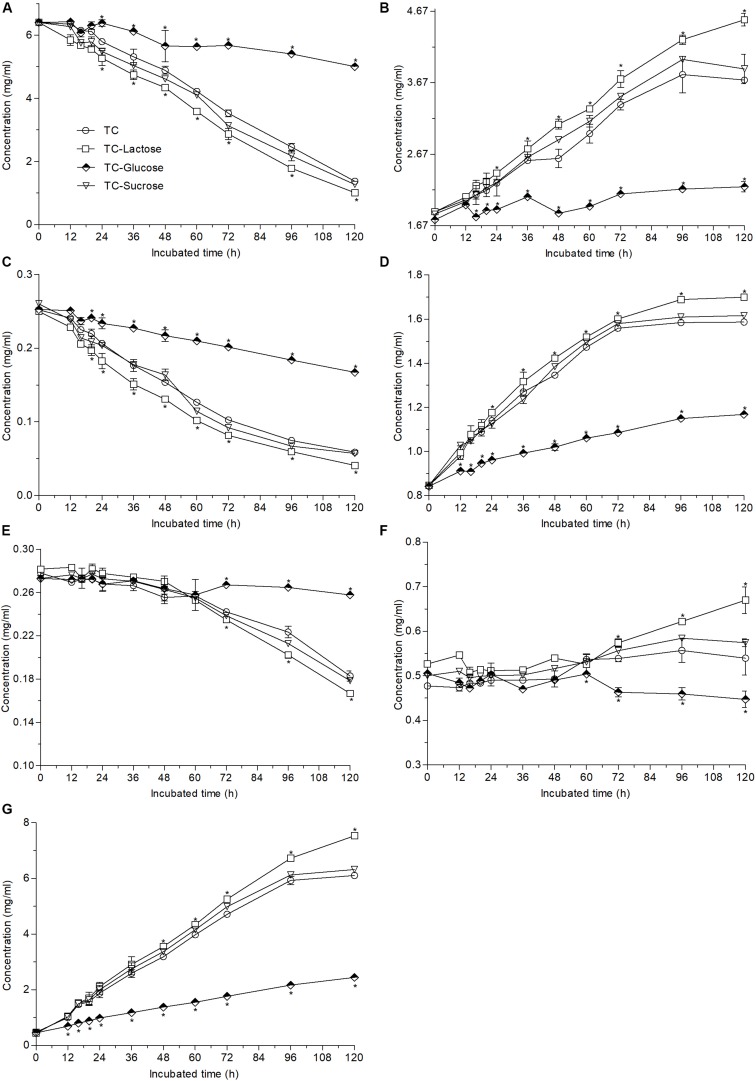
Changes in the contents of EGCG **(A)**, EGC **(B)**, ECG **(C)**, EC **(D)**, GCG **(E)**, GC **(F)**, and GA **(G)** caused by the addition of different carbon sources (60 g/L) into 12 g/L green tea catechins co-incubated with *A. niger* RAF106 for 120 h. Asterisked bars in each bar group differ significantly from those unmarked (Tukey’s HSD, *p* < 0.05).

When different carbon sources were added into TC medium, the contents of EGCG, ECG, and GCG decreased by 84.3, 83.1, and 40.8% in lactose medium, respectively, by 21.9, 33.8, and 5.5% in glucose medium, respectively, and by 80.1, 78.1, and 34.6% in sucrose medium, respectively (Figures 4A,C,E), meanwhile the change trends of non-ester-catechins and GA were nearly the same as those of ester-catechins after 120-h fermentation (Figures 4B,D,F,G), which suggested that compared with the decreases by 79.1% for EGCG, 76.7% for ECG, and 34.2% for GCG, respectively, in TC medium, lactose could improve biotransformation efficiency of ester-catechins, but glucose inhibited the biotransformation (*p* < 0.05), while sucrose had slightly positive effects on the biotransformation.

Moreover, the strongest accelerator and inhibitor were chosen to assess whether the changes caused by different carbon and nitrogen sources are dependent on their concentrations. Compared with the time needed for complete degradation of ester-catechins in the TC medium supplemented 1 g/L yeast extract, the time increased by 8 h for EGCG and 4 h for ECG, respectively, in the TC medium with 0.5 g/L yeast extract, but decreased by 4 h for each ester-catechins in the medium with 5 g/L yeast extract (Supplementary Figures S2A–C). Meanwhile, the more glucose added into TC medium with 1 g/L yeast extract resulted in the slower of the biotransformation (Supplementary Figures S2D–F). The findings suggested that yeast extract accelerated the biotransformation of green tea catechins while glucose inhibited the biotransformation in a dose-dependent manner.

These results demonstrated compared with the biotransformation in the TC medium, nitrogen sources obviously accelerated the degradation of ester-catechins as well as non-ester-catechins and GA, where yeast extract was the strongest promoter, followed by peptone and NaNO_3_ or NH_4_Cl, but different carbon sources exhibited different effects on the biotransformation of ester-catechins, where lactose significantly promoted the degradation while glucose obviously inhibited the degradation.

### Changes in the Antioxidant Capacity, Tannase Activities, and Transcriptional Expression of Mono-/di-oxygenases Caused by Different Nutrients

**FIGURE 5:**
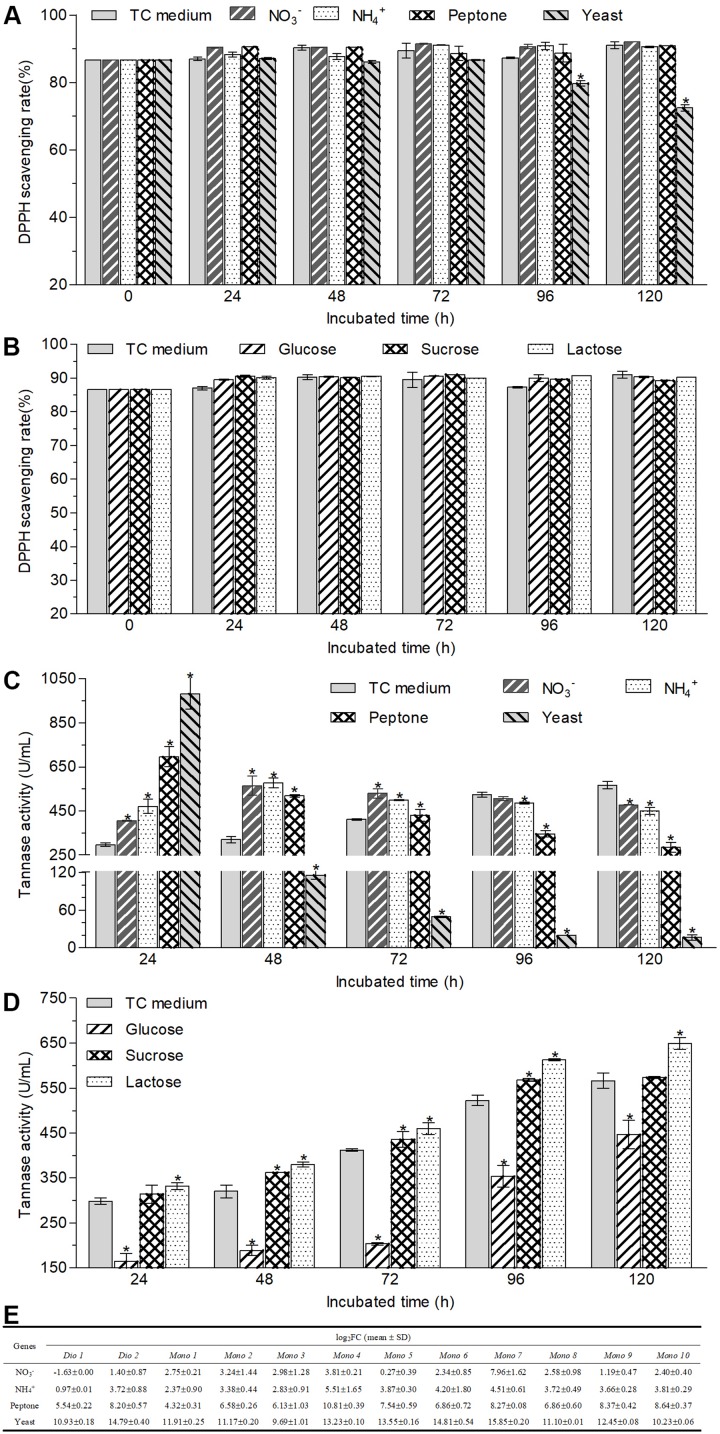
Changes in the antioxidant capacities **(A,B)** and tannase activities **(C,D)** of the supernatant of cultural broth and the transcriptional expression of tested mono-/di-oxygenases **(E)** in the hyphal cells after *A. niger* RAF106 cultivated in the media with different nitrogen **(A,C)** and carbon sources **(B,D)**. Antioxidant capacities were assayed by DPPH, and the tannase activities were tested according to the spectrophotometric method of methanolic rhodanine. The relative transcription level of each gene was defined as the fold changed ratio (FC) of its transcript of 48-h-old hyphal cells obtained from TC medium with a certain nitrogen source over that from TC medium using the 2^–ΔΔCt^ method. Asterisked bars in each bar group differ significantly from those unmarked (Tukey’s HSD, *p* < 0.05).

The changes in the antioxidant capacity of transformed green tea catechins caused by different carbon and nitrogen sources were determined by DPPH assay. As shown in Figures 5A,B, except yeast extract, different supernatant sources obtained from different nitrogen and carbon cultures exhibited equivalent DPPH scavenging rate with those of the supernatant sources of TC medium and non-transformed green tea catechins during 120-h incubation. For the supernatant from the cultures containing yeast extract, the estimate was similar to those of other cultures during 72-h incubation but began to drop from 96 h, and decreased by 16.4% at the 120-h incubation.

Moreover, tannases have been inferred to be important enzymes for the biotransformation of ester-catechins into non-ester-catechins in *A. niger* RAF106 (Figure 2D), so tannase activities were assayed under different carbon and nitrogen sources to find the potential explanation for the changes in the biotransformation of green tea catechins caused by different carbon and nitrogen sources. For different nitrogen sources, the total activities of tannase increased by 229.3% in TC medium with yeast extract, 133.2% in TC medium with peptone, 36.1% in TC medium with NaNO_3_, and 58.0% in TC medium with NH_4_Cl, respectively, compared with that in TC medium after fermentation mediated by *A. niger* RAF106 for 24 h (Figure 5C). As the fermentation time went, the tannase activities in cultures with yeast extract and peptone drop significantly all along, while the tannase activities in cultures with NaNO_3_ and NH_4_Cl increased gradually till 48th hour reached the maximal, and then declined slowly. After green tea catechins were incubated with *A. niger* RAF106 for 120 h, the tannase activities decreased by 97.1% in cultures with yeast extract, 49.5% in cultures with peptone, 15.5% in cultures with NaNO_3_, and 20.6% in cultures with NH_4_Cl, respectively, compared with that in cultures without any extra nitrogen source (Figure 5C). But for different carbon sources, tannase activities increased gradually increased as the fermentation time went, and the estimates in cultures with glucose were lower but those in cultures with lactose were higher than those in TC medium all the time (Figure 5D). At 120-h fermentation, the total activities increased by 14.7% for lactose but decreased by 21.1% for glucose, while sucrose had no significant effect on tannase activity but slightly raised tannase activity by 5.8–13.1% from 48- to 72-h fermentation (Figure 5D). The findings indicated that the changes in tannase activities caused by different nitrogen and carbon sources were in agreement with the changes in ester-catechins triggered by different sources, which might explain why different nitrogen and carbon sources affected the biotransformation of ester-catechins into non-ester-catechins mediated by RAF106, and declines in tannase activities at the late fermentation stages caused by different nitrogen sources suggested that tannases might not be indispensable for the biotransformation of non-ester-catechins and GA.

Furthermore, oxygenase enzymes are important for the opening of the catechol rings, benzene rings and phenolic rings, so the transcriptional expression of 12 mono-/di-oxygenases was assayed under different nitrogen sources to find potential explanation for the degradation of GA and/or non-ester-catechins caused by different nitrogen sources. The result showed that the transcript of 12 mono-/di-oxygenases was significantly enhanced by 821–59063 folds for yeast extract, 19–1794 folds for peptone, 0.95–45 folds for NH_4_Cl, and 0.21–248 folds for NaNO_3_ except *Dio 1*, respectively (Figure 5E). These data suggested that GA and/or non-ester-catechins might be further degraded by mono-/di-oxygenases into smaller molecules in the TC medium with extra nitrogen sources.

## Discussion

Green tea catechins accounting for 75–80% of tea soluble ingredients are the major polyphenols responsible for tea bioactivities such as antioxidant, antimutagenic, anti-cancer, and so on (Wang et al., 2003; Shimizu et al., 2011), which could be improved by biotransformation mediated by enzymes and microbes (Lu and Chen, 2008; Zhong et al., 2008a, b; de Lacey et al., 2014; Ni et al., 2015). Here, *A. niger* RAF106 isolated from the Pu-erh tea could degrade ester-catechins including EGCG, ECG and GCG into non-ester-catechins and GA (Figures 1A–C and Supplementary Figure S1), which was consistent with the previous findings on the biotransformation mediated by the whole-cell microbes (Jayabalan et al., 2007; Qin et al., 2012; Chen and Sang, 2014; de Lacey et al., 2014), but was the first reported in the media only containing green tea catechins, which suggested that *A. niger* RAF106 could be applied in the tea processing with the poor raw material. The yield of EGC, EC, and GC suggested that bioavailability of biotransformed green tea catechins might be higher than that of original green tea catechins because EGC, EC, and GC harbor higher bioavailability than their corresponding ester-catechins (EGCG, ECG, and GC, respectively) which are main components in original green tea catechins (Xu et al., 2004; Peters et al., 2010). Moreover, supplementation with different nitrogen sources shortened the time needed for the full transformation of ester-catechins (Figure 3), and especially for yeast extract, the time was obviously less than those reported in *A. niger* (Accession No. EU314996) from the fermentation bulk of ripened Pu-erh tea during solid-state fungal fermentation (Qin et al., 2012). Meanwhile, non-ester-catechins and GA were still degraded after long-term fermentation, which was also found in the biotransformation of fresh tea leaves of *C. assamica* during fermentation with different in *A. niger* and *Aspergillus fumigatus* (Qin et al., 2012). Except for nitrogen sources, lactose also accelerated the conversion of ester-catechins into non-ester-catechins and GA (Figure 4). However, the conversion was suppressed by glucose in a dose-dependent manner (Figure 4 and Supplementary Figure S2) even though glucose promoted the hyphal growth of *A. niger* RAF106 (Supplementary Figure S3A).

Besides, biotransformed catechins or tea extract mediated by the whole-cell microbes such as *Bifidobacterium animalis* ssp. *lactis* LAFTI^®^ B94, and tannases from different fungi could possess higher antioxidant properties than original catechins or tea extracts, but *Trichoderma* spp. reduced the antioxidant capacities of Assam tea leaves after solid-state fermentation (Macedo et al., 2011; de Lacey et al., 2014; Liu et al., 2017; Kritsadaruangchai et al., 2019). Here, after green tea catechins were incubated with *A. niger* RAF106 in different nutrient media except yeast extract for different hours, the DPPH scavenging rates of different supernatant sources were equivalent to that of original green tea catechins (Figures 1D, 5A,B), which could indicate that the products formed from green tea catechins could have the same or similar antioxidant activity as green tea catechins. But when the media were added with yeast extract, the DPPH scavenging of biotransformed green tea catechins after 96-h fermentation decreased gradually (Figure 5A), which might be due to the degradation of most phenol compounds into non-phenol compounds without any antioxidant capacity in the media containing yeast extract.

Furthermore, it was found that extracellular enzymes in *A. niger* RAF106 were induced by green tea catechins to degrade ester-catechins into non-ester-catechins and GA (Figures 2A–C). Meanwhile, tannase activities in the crude extracts of extracellular enzymes showed tannase might be a core enzyme during the degradation of ester-catechins into non-ester-catechins and GA in *A. niger* RAF106, which was agreement with the previous studies (Macedo et al., 2012; Ni et al., 2015; Liu et al., 2017; Ong and Annuar, 2017), and the tannases involved in the biotransformation of green tea catechins were inducible enzymes as the extracellular form, which coincided with reported conclusion that most microbial tannases are induced enzymes as the extracellular form and the tannases/hydrolases from *Paecilomyces variotii* and *A. niger* which could convert ester-catechins into non-ester-catechins and GA (Aguilar et al., 2001; Zhong et al., 2008b; Macedo et al., 2011, 2012; Yao et al., 2014; Govindarajan et al., 2016; Aharwar and Parihar, 2018). In addition, most microbial tannases greatly influenced by nutritional factors such as nitrogen and carbon sources (Aguilar et al., 2007; Yao et al., 2014), which triggered us to investigate whether change in tannase activities was one critical factor leading to different responses for *A. niger* RAF106 to different nitrogen and carbon sources during the biotransformation of green tea catechins. As expected, yeast extract, peptone, NaNO_3_, and NH_4_Cl improved the tannase activities at the early stage of fermentation (Figure 5C), and lactose elevated but glucose inhibited the tannase activity during the whole fermentation (Figure 5D), which was in well agreement with the changes in biotransformation of ester-catechins into non-ester-catechins caused by different nitrogen and carbon sources, indicating change in tannase activities was one critical factor for the regulation of biotransformation of green tea catechins mediated by *A. niger* RAF106, and was in accordance with the previous studies that NaNO_3_ and NH_4_Cl increase the production of tannases and glucose above 1% concentration inhibits the tannase production, but was contrary to the previous study that yeast extract decreases the tannase production (Bradoo et al., 1997; Raghuwanshi et al., 2011; Sivashanmugam and Jayaraman, 2011). However, the dramatic declines of tannase activities in yeast extract and peptone samples at the late stage of fermentation might be due to the dramatic changes in pH which was not affected significantly by different carbon sources (Supplementary Figures S3B,C) and/or the non-correlation between tannase and the degradation of non-ester-catechins and GA at the late stage of fermentation. Moreover, P450 monooxygenases are involved in aromatic compound degradation, and dioxygenases including extradiol and intradiol dioxygenases catalyze the cleavage of catechol/phenolic/aromatic rings (Contreras-Domínguez et al., 2006; Roopesh et al., 2010; Wadke et al., 2016; Córdova et al., 2017; Kawabata et al., 2019). Meanwhile, non-ester-catechins from the degradation of ester-catechins could be metabolized by gut microbiota to produce the ring fission metabolites (γ-valerolactones), which further degrade to form lower molecular weight phenolic acids (Takagaki and Nanjo, 2010; Chen and Sang, 2014). Here, compared with TC medium, all of 12 mono-/di-oxygenases including intradiol and extradiol dioxygenases and P450 monooxygenases were significantly up-regulated except *Dio 1* (intradiol dioxygenases) for NaNO_3_ in TC medium with different nitrogen sources (Figure 5E), suggesting nitrogen sources might further promote the degradation of GA and/or non-ester-catechins into the ring fission metabolites and smaller molecules through up-regulating the expression of mono-/di-oxygenases after ester-catechins were degraded into non-ester-catechins and GA by tannases. Lastly, all tested nitrogen and carbon sources promoted the hyphal growth of *A. niger* RAF106 (Supplementary Figure S3A), but the increased amount seemed not to perfectly fit what happened to the changes of biotransformation caused by different nitrogen and carbon sources, suggesting the increases in hyphal growth was another explanation but not the most important one for acceleration of biotransformation caused by different nitrogen and carbon sources.

## Conclusion

*Aspergillus niger* RAF106 isolated from Pu-erh tea is capable of growing and mediating the degradation of ester-catechins into non-ester-catechins with higher bioavailability in the media only containing green tea catechins and the biotransformation process is inducible. Our results exhibited the dynamic changes of hydrolyzing green tea catechins (i.e., EGCG, ECG, GCG, EGC, EC, and GC) and GA mediated by *A. niger* RAF106, and the hydrolysis was accelerated by nitrogen sources and lactose through mediating hyphal growth and tannase activities but decelerated by glucose through suppressing tannase activities. Furthermore, nitrogen sources also promoted the degradation of GA and/or non-ester-catechins by significantly up-regulating the transcriptional expression of mono-/di-oxygenases, which was never reported in the previous studies. Additionally, biotransformed green tea catechins in the TC medium with or without different nutrient sources except yeast extract were qualified with equivalent antioxidant capacities to original green tea catechins during the whole incubation, but the further degradation of non-ester-catechins and GA caused by yeast extract resulted in the decrease of antioxidant ability. These findings supposed that *A. niger* RAF106 could be used to manufacture green tea catechins with a specific proportion of active compounds and decided the appropriate time during the period of fermentation to get the high contents of certain simple catechin (i.e., EC and EGC) and GA. Further research will be necessary to study the metabolites formed by the fungus from each compound in order to draft the biotransformation pathways of green tea catechins and determine the cause of the inconstant antioxidant activities.

## Data Availability Statement

All datasets generated for this study are included in the article/Supplementary Material.

## Author Contributions

XF contributed to manuscript revision and overall support of this study. MD and TL performed the whole experiments and analyzed the data. QF, JC, and XM assessed the antioxidant capacities by DPPH assay. ZL, QZ, and SZ contributed to monitoring the changes of green tea catechins. JW designed and performed the experiments, analyzed the data, and prepared the manuscript.

## Conflict of Interest

The authors declare that the research was conducted in the absence of any commercial or financial relationships that could be construed as a potential conflict of interest.
