# Supplementary material for: Changes in the Biotransformation of Green Tea Catechins Induced by Different Carbon and Nitrogen Sources in Aspergillus niger RAF106
Source: Front Microbiol. 2019 Nov 1;10:2521. doi: 10.3389/fmicb.2019.02521 (PMC6839139; doi:10.3389/fmicb.2019.02521)
Supplement: Supplementary file 1 [file Data_Sheet_1.pdf]

Supplementary material

Journal: Frontiers in Microbiology

Submission date: 24 July, 2019

Re-submission date: 15 September, 2019

Changes in biotransformation of tea catechins induced by different carbon and nitrogen sources in *Aspergillus niger* RAF106

Xiang Fang[†], Minru Du[†], Tong Liu[†], Qian'an Fang, Zhenlin Liao, Qingping Zhong, Jianwen Chen, Xiaolin Meng, Shiyu Zhou, Jie Wang[*]

Guangdong Provincial Key Laboratory of Nutraceuticals and Functional Foods, College of Food Science, South China Agricultural University, Guangzhou 510642, China

[†]These authors contribute equally to the study.

[*]**Corresponding author.** E-mail: wangjielangjing@126.com

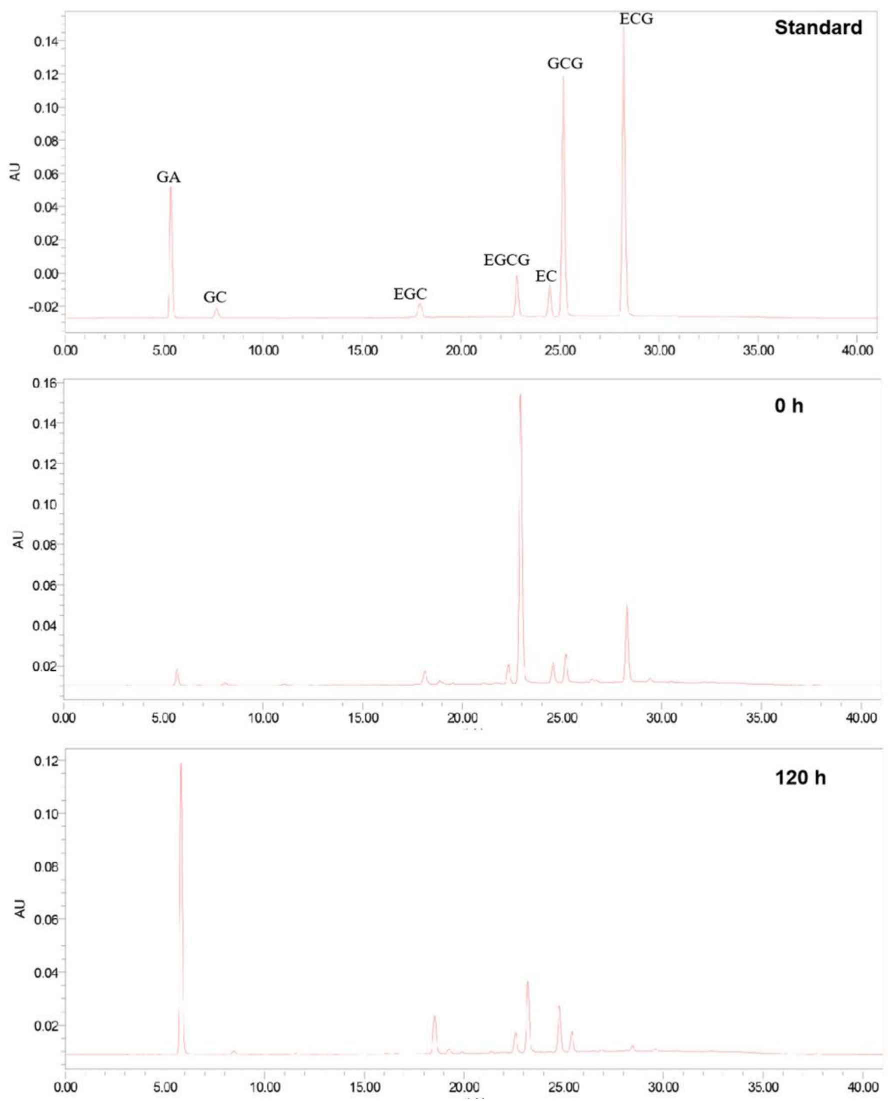

Fig. S1 Chromatograms of standards and the changes in the contents of main components in tea catechins before (0 h) and after (120 h) tea catechins were incubated with *A. niger* RAF106. The standard solution and the surpernatant sources from fermented cultures were filter with a 0.45 μm pore filter and then were injected into an HPLC system.

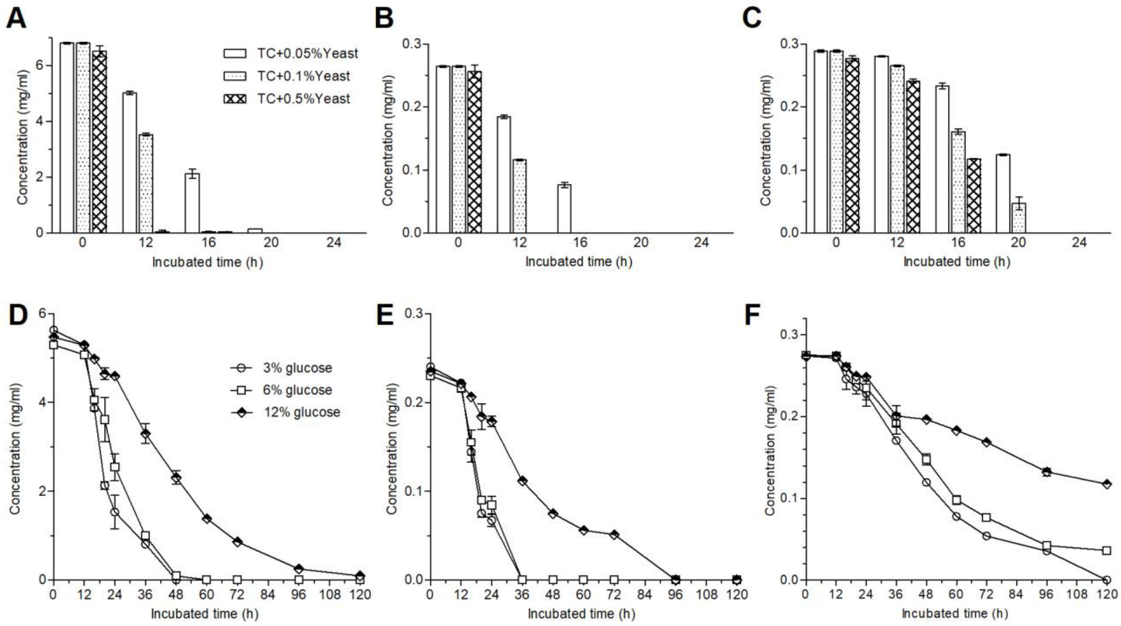

Fig. S2 Effects of yeast extracts and glucose at different doses on the biotransformation of tea catechins mediated by *A. niger* RAF106. (A) and (D) Changes in the contents of EGCG during fermentation for 120 h. (B) and (E) Changes in the contents of ECG during fermentation for 120 h. (C) and (F) Changes in the contents of GCG during fermentation for 120 h.

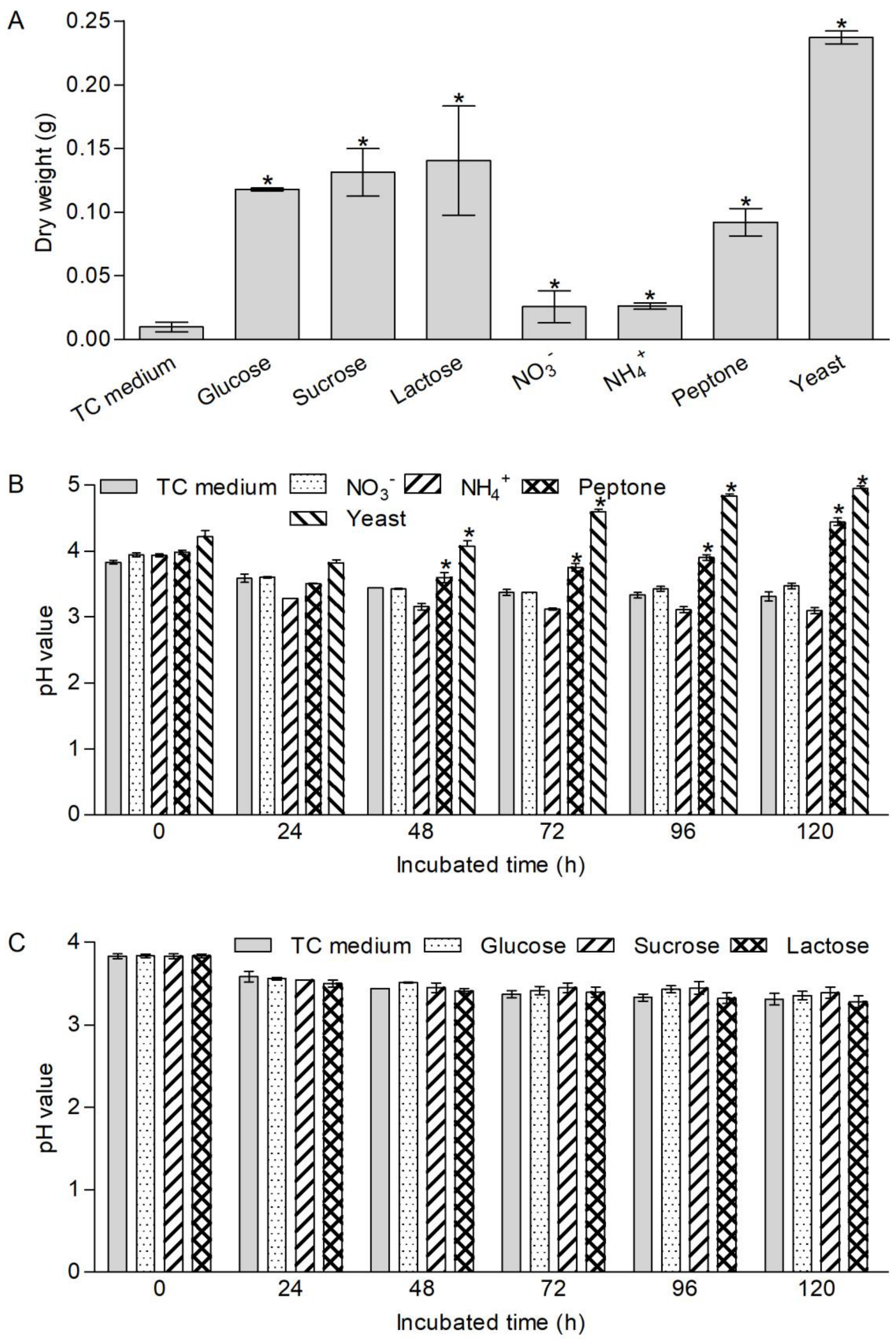

Fig. S3 Dry weight of *A. niger* RAF106 hypha after cells were cultivated in TC medium supplemented with different nitrogen and carbon sources for 120 h (A), and pH values of cultures when *A. niger* RAF106 was cultivated in TC medium supplemented with different nitrogen (B) and carbon sources for different hours. Asterisked bars in each bar group differ significantly from those unmarked (Tukey's HSD, $P < 0.05$).

Table S1. Sequences of the primers used for qRT-PCR.

Genes	Annotation	Gene ID	Primer sequence (5′–3′)
Dio 1	Intradiol dioxygenases	4979346	CTCGACATAGGAGTTCTG / GTGAATGAGGAGTAGGAG
Dio 2	Extradiol dioxygenase	4980195	GAGAGGACGAACTGGAT / CCGACTGTGGAATACATAC
Mono 1	Monooxygenase	10098209	CTCCTCTACAGAACCATC / GAAGACCACGATAGACTC
Mono 2	Monooxygenase	4978054	CTCTGGCTCTTCTTCTTC / TAGTAGCGAGGGTAGTAG
Mono 3	P450 monooxygenase	4985558	ATCTACTGCCTCTACCTC / GAGTGCTTCCTTGATGAC
Mono 4	P450 monooxygenase	4989570	CTCTCCACAGAACTATACC / CAGAATGAGGACGATCTC
Mono 5	P450 monooxygenase	4987242	CTACGGAGTGAACTGAAG / CCATAGGATAAACGGAGAG
Mono 6	P450 monooxygenase	4987167	GACTGGTTGACCTCTTAC / GAGTAGATACCACGATAGG
Mono 7	P450 monooxygenase	4984530	CTATTGGATGGTGCTCTAC / GAATAGATTCGGAGGAGAC
Mono 8	P450 monooxygenase	4986854	CTAGAGGAAGCAGTCAAG / CAGTATGTGGTGGTTCTC
Mono 9	P450 monooxygenase	4983241	GCCTGATACTCTTCTGAG / GACACTTGGTCTCTGATC
Mono 10	P450 monooxygenase	4984344	CTACTGAGAAGGATAGGAC / CTCTTCCCTGATGATAGG
Actin	Actin		CCACGAGACCACCTTCAACTCC / CCACCGATCCAGACGGAGTACTTGC
